# Inflammation in the Mucous Membrane of the Alimentary Canal, Occasioning Obstinate Diarrhœa

**Published:** 1815-07

**Authors:** James Jackson


*For the Medical and Physical Journal.*

*Inflammation in the Mucous Membrane of the Alimentary Canal, occasioning obstinate Diarrhœa;* by JAMES JACKSON, M.D.

R. G. Esq. a gentleman under thirty, of sanguine temperament, but with a constitution not firm, travelled in the summer of 1813 to Fort George, thence across the country to Albany, and from there to Boston. He began to be sick at Fort George, and from that time was occasionally troubled with diarrhœa, and with some other symptoms of indisposition. He was not, however, much delayed in his journey by these complaints. He grew more sick, so that some days before he reached Boston he was obliged to go to bed in the day time, and at Northampton he took some rhubarb. Yet he was not prevented from riding every day, nor even from exerting himself in visiting whatever seemed worthy his observation on the road. He arrived in Boston on the 15th of September, and the next day I visited him, and found him confined to his bed.

At this time his symptoms were head-ach, pains in the neck and back, muscular debility, pulse rather tense and more than 100, tongue dry and a little coated, appetite diminished, and thirst. The diarrhœa had ceased since taking the rhubarb about three or four days before. He now got a pill of submuriate of quicksilver, and this was followed by a saline cathartic. The operation was kind; and at night he had a small dose of opium, with an antimonial. The next day (17th) the pains were gone, and he seemed better, but toward night the diarrhœa came on. The patient stated that he was prone to this affection, and that he had once been greatly reduced by it. From this time the diarrhœa continued until his death. At first the evacuations were frequent, but after two or three days, they were not more than three or four in twenty-four hours. In quantity they varied from one gill to three. They consisted of a light yellow fluid, thin, but not watery; and had light portions of fæcal matter floating in them. These evacuations took place without pain; and at other times also the patient declared himself free from pain, except very rarely he complained of a momentary uneasiness from flatulence. He was, was, likewise, free from nausea, except once, when it was attempted to give him ipecacuanha in small doses, combined with other articles. The tongue was very dry, but not coated after the first day or two, until the last three days. The thirst was, at all times, very great. There was not any time when the appetite was wholly lost, or at least the patient was always ready to take food. The muscular strength was great from after the first day or two until near the close of the disease. Until within thirty-six hours of his decease, the patient could walk about the room perfectly well. The pulse were mostly at 100 previous to the last three days. When they grew more frequent, they also grew weaker; but at the same time the tongue grew more moist and natural, the desire for food increased, he began to take freely of strong beef-tea, which suited him very well, and the sensations about his stomach were, as he repeatedly and emphatically stated, precisely those of health.

The remedies employed were opiates, antacids, astringents, and tonics, with warm bathing and friction. These remedies did not produce the desired effect; yet the general strength of the patient was preserved, and the disease seemed to be controled, though not conquered.

The termination of the disease and of life was preceded by symptoms of great violence. These were three stools, amounting to nearly or quite two pints, consisting almost entirely of blood. With these the patient immediately began to sink, and he died in thirty-six hours after the first of these discharges, and about twenty-four hours after the last of them. Before death he had a large alvine discharge, in which there was extremely little blood. Death took place on the 28th of September.

*Examination of the Body.*—The abdomen only was opened. On viewing the viscera of this cavity *in situ naturali*, the following circumstances were noticed. The omentum was very red, but entirely without effusion of any kind either within or upon its coats. The small intestines generally were of a light red colour; apparently from a fulness of the extreme vessels immediately under the peritoneal coat. That coat itself was natural, having its usual colour and polish. The gall-bladder was very full, and in shape something different from usual, looking like a portion of small intestine when distended. Its duct, as well as those connected with it, was pervious; and its contents were natural. The liver, spleen, and pancreas, appeared, in all respects, healthy.

The alimentary canal was laid open through its whole extent, excepting the gullet. Its contents were not unnatural.

They were not offensive in smell. In the stomach they consisted of the fluids recently swallowed. In the small intestines they were of the usual consistence, generally of a light yellow colour, but in one part a little green. The quantity in the small intestines was very considerable. The large intestines were proportionably more empty, and their contents were more fluid, and of a more brown colour.

On the internal surface of the stomach and bowels, there were very strong marks of disease. These were seen in the stomach from the cardiac orifice over every part of that organ to about its middle, and in the first part of the duodenum; and more slightly in many parts of the small intestines. The disease was again strongly marked in the ileum, in the four inches of its lower part; in the valve of the colon, and in the first and last parts of the colon; but not in its arch, nor in the rectum.

These marks of disease consisted, for the most part, in a great discolouration, the colour being in some parts of a dark red, and in others quite purple. There was not any swelling, nor any loss of polish in the mucous membrane of the parts discoloured, with the exceptions which follow. In the ileum, at its termination, and in the valve, the mucous membrane was considerably thickened, the surface was rough, and was covered with adhesive mucus. In the large intestines there were, in addition, many small ulcerations; and in some parts, particularly in the rectum, there were many small white points, which were evidently elevated, and had the appearance of very minute pimples. The mucous membrane was not thickened in any part of the large intestines, except the slight swelling and induration at the margin of the little ulcers. These margins were of a bright red colour, while that of the ulcerated surfaces was white or cineritious.

*Reflections.*—The mode in which this case terminated, and the appearances after death, were not such as the symptoms previous to the last days authorised us to expect. One conversant with pathological anatomy, in reading the examination of the body without having read the history of the disease, would say, that in this case there must have existed the symptoms of dysentery, accompanied by nausea and vomiting. Especially it would seem to such an one, that there must have been a difficulty in transmitting the contents of the small intestines through the valve of the colon into the large intestines; and that hence the stools must have been small, and preceded by pain. As this was a case which I watched with peculiar assiduity, I knew that the symptoms above mentioned did not occur. I saw almost every discharge charge from the bowels after the patient came under my care. In a single instance, previous to the last two days, there was a very small portion of bloody mucus; and this seemed evidently connected with an irritation just at the *sphincter ani*, brought on by the frequent dejections.

For the absence of nausea and vomiting, and of distress at the stomach, I cannot suggest any cause. There certainly did not seem to be any diminution of either sensibility or irritability during the disease. It is remarkable, that even the appetite was not lost, and that such food as the patient took seemed to undergo digestion. How account for the absence of dysenteric symptoms? The only difference in the morbid appearances of the large intestines, in this case, from those commonly seen in dysentery, are these:—In dysentery, the mucous membrane of the large intestines in some part, and likewise in some cases that of the small intestines, is found, not only changed in colour, but also swollen, and covered more or less thickly with adhesive mucus. In this case, except on the termination of the ileum and on the valve of the colon, the state of this membrane was quite different. Though greatly discoloured, it was rather unusually smooth and polished, exhibiting an appearance of almost preternatural cleanness.

Whence came the sudden and profuse discharge of blood? No doubt from the exhalents of the large intestines. The intestines were carefully examined, and there was nothing to justify a suspicion of the rupture of any vessel. It is remarkable that there was not in the cavity of the intestines, after death, the slightest particle of blood, nor of bloody fluid.—*New-England Journal.*

